# Tailored nurse-led cardiac rehabilitation after myocardial infarction results in better risk factor control at one year compared to traditional care: a retrospective observational study

**DOI:** 10.1186/s12872-018-0907-0

**Published:** 2018-08-15

**Authors:** Halldora Ögmundsdottir Michelsen, Marie Nilsson, Fredrik Scherstén, Ingela Sjölin, Alexandru Schiopu, Margret Leosdottir

**Affiliations:** 1 Department of Coronary Disease, Skåne University Hospital, Inga Marie Nilsson gata 47, Malmö, Sweden; 2 Department of Clinical Sciences Malmö, Faculty of Medicine, Lund University, Box 117, SE-221 00 Lund, Sweden

**Keywords:** Cardiac rehabilitation, Secondary prevention, Acute myocardial infarction, Cardiovascular risk factors, Nurse-led care

## Abstract

**Background:**

Cardiac rehabilitation improves prognosis after an acute myocardial infarction (AMI), however, the optimal method of implementation is unknown. The aim of the study was to evaluate the effect of individually-tailored, nurse-led cardiac rehabilitation on patient outcomes.

**Method:**

This single-centre retrospective observational study included 217 patients (62 ± 9 years, 73% men). All patients attended cardiac rehabilitation including at least two follow-up consultations with a nurse. Patients receiving traditional care (*n* = 105) had a routine cardiologist consultation, while for those receiving tailored care (*n* = 112) their need for a cardiologist consultation was individually evaluated by the nurses. Regression analysis was used to analyse risk factor control and hospital readmissions at one year.

**Results:**

Patients in the tailored group achieved better control of total cholesterol (− 0.1 vs + 0.4 mmol/L change between baseline (time of index event) and 12–14-month follow-up, (*p* = 0.01), LDL cholesterol (− 0.1 vs + 0.2 mmol/L, *p* = 0.02) and systolic blood pressure (− 2.1 vs + 4.3 mmHg, p = 0.01). Active smokers, at baseline, were more often smoke-free at one-year in the tailored group [OR 0.32 (0.1–1.0), *p* = 0.05]. There was a no significant difference in re-admissions during the first year of follow-up. In the tailored group 60% of the patients had a cardiologist consultation compared to 98% in the traditional group (*p* < 0.001). The number of nurse visits was the same in both groups, while the number of telephone contacts was 38% higher in the tailored group (*p* = 0.02).

**Conclusion:**

A tailored, nurse-led cardiac rehabilitation programme can improve risk factor management in post-AMI patients.

## Background

Patients with established coronary artery disease are at high risk for recurrent coronary events and other comorbidities related to cardiovascular disease (CVD) [1]. According to Swedish national registries, 18% of acute myocardial infarction (AMI) survivors suffer a second CVD event in the first year and approximately 50% of major coronary artery disease events occur in those with a previous hospital discharge diagnosis of AMI [2]. Effective secondary prevention (SP), aiming to reduce the impact of the established disease, administered through cardiac rehabilitation (CR), a medically supervised program designed to improve cardiovascular health, can reduce this risk and improve prognosis [3–9]. In the most recent prevention guidelines from the European Society of Cardiology (ESC), SP at specialized prevention centres for patients who have suffered an acute coronary event is given the highest recommendation (IA) [10]. It is advocated that patients are offered comprehensive programmes that focus on CVD risk factor management addressing both somatic and psychosocial factors, with special emphasis on the benefits of exercise-based CR [10].

Currently there is a consensus that SP is an integral part of treatment after AMI. Its core components are well identified, and current guidelines recommend a multidisciplinary approach to an overall CVD risk reduction [10, 11]. An individual, patient-tailored risk reduction programme is generally advocated, and studies have previously suggested that it is more efficient [9, 12, 13] and cost-effective [14]. However, several factors such as the optimal length and the cumulative benefits of various components of the CR programme remain unclear [7, 10, 11]. This provided incentive to reorganize our CR programme towards a prolonged individually tailored nurse-led programme where patients need for consultation with a cardiologist was individually evaluated. The aim of this change was to provide the most appropriate and effective post-AMI care and prioritize cardiologist resources. The study, then, evaluated whether the change benefitted AMI survivors in terms of better CVD risk factor management and lower hospital readmission rates.

## Methods

The study was a single-centre retrospective observational study comparing a tailored nurse-led CR programme to traditional care.

### Study group, data collection and documentation

A total of 217 consecutive patients with AMI admitted to the coronary care unit (CCU) at Skane University Hospital in Malmö during a 14-month period (2013–2015) and who were registered in the Swedish web-system for Enhancement and Development of Evidence-based care in Heart Disease Evaluated According to Recommended Therapies (SWEDEHEART) registry, were included in the study. SWEDEHEART is a nationwide registry that records baseline characteristics, treatments, follow-up and outcome data of consecutive patients with AMI admitted to CCUs in Sweden [15].

SWEDEHEART contains several sub-registries; including a registry for acute care and a registry for CR. The registry for CR only includes patients under 75 years of age, making age ≥ 75 an exclusion criteria in our study [16]. Documentation in SWEDEHEART starts at the time of AMI and continues at two separate follow-up visits at 6–10 weeks and 12–14 months after the index event. Information is collected using standardized forms which includes all parameters listed in Table 1.

**Table 1:** Study parameters, definitions, targets and measurements

Parameter	Definition	Target	Measurement
Smoking	Active smoker yes/no	Smoking cessation	Self-reported
Exercise training	Reaching target for exercise training	Fitness training 20–60 min at least three times a week and muscular resistance training at least two times a week equivalent 12–16 at Borg’s perceived exertion scale [19]	Participation in hospital-based exercise training, verified by hospital records
Physical activity	Reaching target for physical activity	Any physical activity at least 30 min per day corresponding a brisk walk	Self-reported
Overweight	BMI ≥ 25 kg/m2	Weight loss with target BMI <25 kg/m2	Height in m and weight in kg measured at follow-up visits
Hypertension	SBP ≥140 mmHg DBP ≥90 mmHg	SBP < 140 mmHg DBP < 90 mmHg	With a manual sphygmomanometer with subject in sitting position after 5 min of rest
Blood lipids (mmol/L) above therapeutic goal	TC ≥ 4.5	TC < 4.5	Fasting blood samples (plasma): TC, LDL, HDL, TG
	LDL ≥ 1.8	LDL < 1.8	
	HDL ≤ 1.0	HDL > 1.0 (men)	
	HDL ≤ 1.2	HDL > 1.2 (women)	
	TG ≥ 1.7	TG < 1.7	
Cardioprotective medication	ACEi/ARB, β-blocker, antiplatelet- and lipid-lowering medication	Maximum adherence to treatment	Self-reported
Hospital readmission	Readmission due to CVD	Avoidance	Hospital records

*BMI* Body mass index, *SBP* systolic blood pressure, *DBP* diastolic blood pressure, *TC* total cholesterol, *TG* triglycerides, *LDL* low density lipoprotein, *HDL* high density lipoprotein, *CVD* cardiovascular disease

The study group was divided by a timeline. The first half of the included patients (*n* = 105) received traditional care, while the latter half of the patients (*n* = 112) received tailored, nurse-led CR.

### The follow-up visits (applies to both traditional care and tailored nurse-led care)

Work routines within the CR programme at Skane University Hospital at the time of the study were based on the ESC prevention guidelines from 2012 [17]. The CR team consisted of specialized cardiac nurses, physiotherapists, a counsellor and a supervising cardiologist. The rehabilitation team had daily meetings to discuss patient cases. The patient’s follow-up sessions with a nurse were focused on lifestyle, biometric risk factors and medication adherence (see Table 1). Main targets for healthy lifestyles were smoking cessation, physical exercise and diet and weight management in the case of overweight/obesity. Counselling and educational material on healthy food choices were given according to the 2012 Nordic Nutrition Recommendations [18] and the ESC prevention guidelines [17]. Plasma glucose, haemoglobin A1c (HbA1c) and plasma lipids samples were drawn at two seprate nurse visits and analysed at the Clinical Biochemistry department of Skane University Hospital Malmö with accredited methods. Blood pressure was measured (mmHg) with a manual sphygmomanometer after a 5-min rest with patient in sitting position. If blood pressure control or lipid control was inadequate medication was titrated by the nurse, in cooperation with the treating cardiologist. Weight was measured (kg) in light indoor clothing.

At the time of the study the follow-up visits with a physiotherapist included an individual consultation a few weeks after discharge which included an endurance test on a stationary bicycle, followed by a total of eight physiotherapist-led group training sessions at the hospital. Exercise training consisted of organized, individualized exercise-based training containing both fitness training and muscular resistance training equivalent to 12–16 at Borg’s perceived exertion scale [19]. Additional home-based exercise training as well as daily physical activity were promoted. During the follow-up sessions the patients were asked how many days during the last week they had performed any physical activity at least 30 min per day corresponding to a brisk walk.

### Traditional care

Post-AMI traditional care consisted of a follow-up visit with a nurse at 6–10 weeks after hospital discharge. This first follow-up visit included registry data collection and registration in SWEDEHEART. Following was a standard consultation with cardiologist at three months, after which patients were routinely referred to primary care. More complicated cases and patients with persisting symptoms, however, remained in the care of the CR out-patient unit at the hospital. At 12–14 months, all patients had an additional visit with a nurse, the main reason being the second registration in SWEDEHEART but also for controlling risk factors and offering lifestyle counselling as described above.

### Tailored, nurse-led care

Before discharge, patients in the tailored, nurse-led group received a standardized letter explaining the follow-up protocol emphasizing that their primary contacts after hospital discharge would be a nurse and physiotherapist. The letter also explained that their need for a cardiologist consultation would be evaluated by the nurse. Furthermore, the letter stated that the patients would remain in the care of the CR team at the hospital until the second nurse visit at 12–14 months, after which patients would be referred to primary care.

As in traditional care all patients in the tailored group were offered a first follow-up visit with a nurse at 6–10 weeks after hospital discharge. Patients with remaining significant stenosis after culprit intervention, reduced left ventricular function (ejection fraction < 35%) as measured by echocardiography at the time of index event, remaining or recurring symptoms after the index intervention, and having undergone coronary artery bypass grafting (CABG) were automatically scheduled for a consultation with a cardiologist at approximately 3 months post-AMI. Also, patients who did not fit the criteria but either themselves asked for a follow-up visit with a cardiologist or the nurse assessed such a need were also scheduled for a cardiologist consultation.

Unlike traditional care, no patient was referred to primary care until after the second follow-up nurse visit at 12–14 months. Patients were encouraged to contact the CR team at the hospital if they had any questions concerning their heart disease or in the case of new cardiac symptoms – issues which in the traditional group were routinely referred to primary care after the cardiologist consultation at three months post-AMI. In addition, all patients in the tailored, nurse-led group received a letter from the nurse at approximately 6–8 months, promoting healthy lifestyles. At the same time new laboratory measures of lipids and blood glucose were performed. In the case of deranged laboratory values, patients were subsequently contacted by a nurse by telephone.

### Information and consent

In accordance with Swedish law, all patients are informed verbally about data registration in the SWEDEHEART registry and the right to get their data erased from the registry upon request. During the 14-month study period none of the patients that were included in the study requested to get their data erased from the registry.

The Regional Ethical Review Board at Lund University approved the study (Dnr 2016/494).

### Follow-up and outcome

The pre-specified primary outcomes were comparative delta values for continuous risk factors obtained at the first and second follow-up visits with the nurse. These included systolic and diastolic blood pressure, body mass index (BMI), low density lipoprotein (LDL), high density lipoprotein (HDL) and total cholesterol and triglycerides. Smoking and self-reported physical activity, were compared by direct measures performed at the 12–14-month visit.

Secondary outcomes were the number of follow-up visits and telephone consultations with a nurse or cardiologist and hospital re-admittance rates during the first year post-AMI.

### Statistical methods

Baseline characteristics were described with means (± standard deviations) and percentages, using independent samples T-test and chi-square test to assess significance.

A multivariable linear regression analysis using backward selection was used to compare outcome measures between the groups for continuous variables, adjusting for age, gender, BMI, participation in an exercise training programme, comorbidities including previous coronary heart disease and diabetes mellitus, and cardioprotective medication (platelet inhibitors, Angiotensin converting enzyme inhibitors (ACEi), Angiotensin II receptor blockers (ARBs), β-blockers, statins and ezetimibe). Logistic regression analysis, adjusting for the same variables, was used to compare outcome measures between groups for categorical variables. To minimize the confounding effect of any differences between the groups at baseline, for continuous variables, delta values were calculated between the first and second follow-up rather than comparing crude measurements at 12–14 months only. However, as the 6–10-week variable for self-reported physical activity was confounded by the fact that the patients in the tailored nurse-led group more frequently participated in hospital-based exercise training at that time, the actual 12–14-month follow-up variable was used (not delta). All data was analysed by using the SPSS 23.0 statistical software package (SPSS Inc., Chicago, IL).

## Results

### Baseline characteristics

Baseline characteristics (at the time of the index event) are shown in Table 2. No patient was lost to follow-up. Except for baseline total cholesterol, there were no significant differences between the groups.

**Table 2:** Baseline characteristics

	Traditional care	Tailored nurse-led care	*P*-value
Number of patients:	105	112	
Demographics:			
• Men	*n* = 57, 71%	*n* = 83, 74%	0.67
• Age, years	61.7 (±9.1)	61.8 (±8.0)	0.10
Risk factors:			
• Diabetes Mellitus	*n* = 33, 31%	*n* = 21, 19%	0.10
• Active smoker	*n* = 43, 41%	*n* = 40, 36%	0.35
• SBP, mmHg	149.9 (±32.2)	153.7 (±24.2)	0.33
• DBP, mmHg	87.1 (±17.3)	87.4 (±15.2)	0.90
• BMI, kg/m2	27.3 (±4.5)	27.9 (±4.5)	0.37
• Total cholesterol, mmol/L	5.0 (±1.2)	4.6 (±1.0)	0.01
• LDL, mmol/L	3.0 (±1.2)	2.7 (±0.9)	0.45
• HDL, mmol/L	1.2 (±0.4)	1.2 (±0.4)	0.19
• TG, mmol/L	1.8 (±1.3)	1.5 (±0.7)	0.07
LVEF at the time of index event			
• Normal (≥50%)	*n* = 68, 65%	*n* = 88, 79%	0.09
• Mildly decreased (40–49%)	*n* = 20, 19%	*n* = 17, 15%	
• Moderately decreased (30–39%)	n = 8, 8%	n = 4, 4%	
• Severely decreased (≤29%)	n = 3, 3%	n = 0	
History of cardiovascular disease:			
AMI, PCI or CABG	*n* = 19, 18%	*n* = 22, 20%	0.83
Type of myocardial infarction			
• NSTEMI	*n* = 74, 70%	*n* = 73, 65%	0.40
• STEMI	*n* = 31, 30%	*n* = 38, 34%	0.49
Number of vessels affected			
• No significant stenosis	n = 7, 16%	n = 4, 10%	0.44
• 1–2 affected vessels	*n* = 23, 51%	*n* = 27, 64%	
• 3 affected vessels or left main stenosis	*n* = 15, 33%	n = 11, 26%	
In-hospital treatment:			
• PCI	*n* = 81, 77%	*n* = 85, 76%	0.62
• CABG	*n* = 12, 11%	n = 20, 18%	0.25
Pharmacological treatment at hospital discharge:			
• Platelet inhibitors	n = 105, 100%	*n* = 111, 99%	0.33
• Statins	*n* = 104, 99%	*n* = 110, 98%	0.97
• Ezetimibe	n = 1, 1%	n = 1, 1%	0.62
• ACEi or ARB	n = 103, 98%	n = 103, 92%	0.06
• Β-blockers	*n* = 91, 87%	*n* = 96, 86%	0.62

Numbers presented as numbers (n) and percentages (%) or means (±SD) and *p*-values for difference

*SBP* systolic blood pressure, *DBP* diastolic blood pressure*, BMI* body mass index, *LDL* low density lipoprotein, *HDL* high density lipoprotein, *TG* triglycerides, *LVEF* left ventricular ejection fraction, *PCI* percutaneous coronary intervention, *CABG* coronary artery bypass grafting, *NSTEMI* non-ST elevation myocardial infarct, *STEMI* ST elevation myocardial infarct, *ACEi* angiotensin converting enzyme inhibitor, *ARB* angiotensin II receptor blocker

Patients in the tailored group had a higher participation rate in hospital-based exercise training during follow-up (74% vs 61%, *p* = 0.04). Self-reported use of cardioprotective medication at one year was similar between the two groups apart from a higher reported intake of ezetimibe in the tailored group compared to the traditional group (21% vs 10%, p = 0.04) (see Table 3).

**Table 3:** Pharmacological treatment and participation in exercise training reported at the 12–14-month visit

	Traditional care	Tailored nurse-led care	*P*-value
Pharmacological treatment:			
• Platelet inhibitors	*n* = 93, 89%	*n* = 102, 91%	0.54
• Statins	*n* = 92, 88%	n = 102, 91%	0.41
• Ezetimibe	n = 11, 10%	n = 23, 21%	0.04
• ACEi or ARB	n = 91, 87%	n = 96, 86%	0.84
• Β-blocker	*n* = 78, 74%	n = 85, 76%	0.98
Participation in exercise training	*n* = 64, 61%	n = 83, 74%	0.04

Numbers presented as numbers (n) and percentages (%)and p-values for difference

*ACEi* angiotensin converting enzyme inhibitor, *ARB* angiotensin II receptor blocker

### Primary end-points

Results from the multivariable analysis for continuous variables are displayed in Table 4. We observed significant reductions in systolic blood pressure, total and LDL cholesterol between the first and second nurse visits favouring the tailored, nurse-led CR group. Active smokers at baseline were more often smoke-free at the 12–14-month follow-up in the tailored nurse-led care group: *n* = 25 out of 40 (63%) vs. *n* = 18 out of 43 (42%); OR 0.32 (CI 0.1–1.0), *p* = 0.05 whereas self-reported physical activity was lower (1*.3 (±2.1)* vs 1.9 (±2.3) days during the last week performing at least 30 min of moderate physical activity, *p* = 0.04).

**Table 4:** Risk factors at first (6–10 weeks) and second (12–14 months) follow-up visits and the difference (delta values) there between for patients receiving traditional and tailored nurse-led care, respectively

	Traditional care			Tailored, nurse-led care			
	First visit	Second visit	Difference (delta value)	First visit	Second visit	Difference (delta value)	P for difference
SBP (mmHg)	129.2 (±18.5)	133.5 (±21.0)	+4.3 (±20.3)	132.8 (±20.2)	130.7 (±15.1)	−2.4 (±16.0)	0.01
DBP (mmHg)	79.8 (±10.6)	79.9 (±10.3)	+ 0.1 (±11.6)	81.7 (±10.0)	79.1 (±8.9)	−2.6 (±9.3)	0.18
BMI (kg/m^2^)	27.8 (±4.6)	26.2 (±3.8)	−0.5 (±1.5)	30.7 (±2.5)	28.3 (±3.9)	−0.1 (±1.8)	0.15
Total cholesterol (mmol/L)	3.5 (±0.68)	3.8 (±0.98)	+ 0.4 (±1.0)	3.6 (±0.83)	3.6 (±0.93)	−0.1 (±0.8)	0.02
LDL (mmol/L)	1.6 (±0.59)	1.8 (±0.91)	+ 0.2 (±0.9)	1.8 (±0.66)	1.6 (±0.70)	−0.1 (±0.7)	0.02
HDL (mmol/L)	1.2 (±0.40)	1.4 (±0.43)	+ 0.1 (±0.3)	1.2 (±0.41)	1.3 (±0.43)	+ 0.1 (±0.2)	0.06
TG (mmol/L)	1.36 (±0.82)	1.35 (±0.92)	−0.01 (±0.8)	1.35 (±0.75)	1.31 (±0.79)	−0.04 (±0.7)	0.59

Numbers presented as means (±SD) and p-values for difference. Adjusted for age, gender, previous heart disease, diabetes mellitus, BMI, participation in exercise training, and cardioprotective medication at 12–14-months

*SBP* systolic blood pressure, *DBP* diastolic blood pressure, *BMI* body mass index, *LDL* low density lipoprotein, *HDL* high density lipoprotein, *TG* triglycerides

### Secondary end-points

In the tailored nurse-led group 34% of patients were scheduled for a follow-up visit with a cardiologist in accordance with the predefined criteria. In addition, 29 patients were, after an individual evaluation by the CR teams nurse, scheduled for a follow-up visit with a cardiologist. Thus, in total, 60% (*n* = 67 out of 112) of the patients had at least one cardiologist consultation during the follow-up period. In the traditional care group 98% of the patients had a scheduled consultation with a cardiologist (*n* = 103 out of 105, p for difference < 0.001). Two patients in the traditional care group preferred their follow-up to be with a privately practicing cardiologist (i.e. not working at the CR unit), both patients having a previous history of CVD. Of all the patients in the tailored care group who were assessed not to be in need for a cardiologist consultation, only one patient asked to see a cardiologist. The number of nurse visits was the same (2.4 visits/patient in the tailored group vs 2.6 visits/patient/year in the traditional group, *p* = 0.30), while telephone contacts with the CR team increased by 38% (5.8 vs 4.1 telephone contacts/patient/year, *p* = 0.02) in the tailored care group.

There was a non-significant trend towards more re-admissions due to cardiovascular causes (angina, AMI, heart failure and stroke) in the traditional group: *n* = 10 out of 105 (9.5%) vs. *n* = 4 out of 112 (3.6%) in the tailored nurse-led group; OR 2.8 (CI 0.8–9.3), *p* = 0.1. Out of readmissions in the tailored nurse-led group all four were due to angina and no patients were readmitted due to AMI, heart failure or stroke (*n* = 0 out of 112 (0.0%) vs. *n* = 8 out of 105 (7.6%) *p* = 0.003).

## Discussion

In this study we compared two groups of AMI survivors who received either traditional post-AMI care or tailored, nurse-led CR. In the tailored group the patients’ main contact at the CR clinic was a nurse and the need to have a post-AMI follow-up visit with a cardiologist was individually evaluated. In addition, a letter from the nurse promoting healthy lifestyles, and laboratory measures of lipids and blood glucose were performed at approximately 6–8 months. Finally, patients in the tailored group were not referred to primary care until after the 12–14-month follow-up visit with a nurse. The prolonged care did not lead to an increased number of visits to a nurse, and cardiologist visits were fewer. However, the number of telephone contacts increased.

We observed a significantly better control of total and LDL cholesterol and systolic blood pressure between baseline and at 12–14-months in the tailored group compared to the traditional group after adjusting for use of cardioprotective medication, prior CVD, diabetes and participation in exercise training. This, in turn, can reduce the relative risk for repeated CVD events [20, 21]. There was also a significant increase in smoking cessation rates at 12–14 months follow-up in the tailored group and a significantly higher participation rate in the hospital-based exercise training programme. The tailored group had less self-reported physical activity, which can be confounded by the higher participation in the exercise training programme. There was a non-significant trend towards fewer re-admissions due to cardiovascular causes during the first year post-AMI in the tailored group. Our findings suggest that offering tailored nurse-led CR may have a beneficial effect on outcomes as compared to the traditional type of care where patients receive a standardized, one-for-all follow-up.

Despite the acknowledged importance of CR, patients are not reaching their therapeutic goals [4]. The ideal method of implementation remains unclear and consequently the exemplary rehabilitation programme has yet to be designed [4, 7, 10, 17, 22, 23]. Suggested reasons for patients not reaching preventive goals include CR programmes commonly relying on short-term interventions, being rigid in design, and not being adequately implemented [4, 24–26]. The reorganization of the CR programme at our clinic was designed to increase programme flexibility and to optimize utilization of the team’s qualifications. As such, the primary patient responsibility lies with the nurses, while cardiologist resources focus on high-risk patients.

The decision of having the CR programme coordinated by nurses was based on evidence that nurse-led programmes can improve lifestyle, risk factor control and quality of life [8, 9, 27, 28]. EUROACTION was a multicentre study that demonstrated significant benefits of a nurse-led CR programme. The study showed that patients who participated in a nurse-led study model showed improvement in risk factor management and lifestyle changes as compared to standard care [9]. Another example is the RESPONSE trial, a study that randomized post-AMI patients to a 6 month CR programme with either traditional- or nurse-led care [8]. At 12–14 months post-AMI patients in the nurse-led group had better risk factor control, fewer hospital readmissions and emergency department visits as well as a 17% lower predicted risk ratio of mortality when compared to the control group. Our study results are in line with EUROACTION and RESPONSE trial results, confirming the benefits and safety of nurse-led SP programmes with an interdisciplinary approach. However, these studies included multiple follow-up nurse visits which requires resources. Our study model is easily applicable since patients, even though, remaining in the care the CR team under a 12–14-month period had two follow-visits with a nurse and the need for a consultation with a cardiologist is individually assessed and, thus, resources were prioritised.

Apart from receiving a nurse-led programme, patients in the tailored group remained in the care of the CR clinic for 12–14 months post-AMI while patients in the traditional group were routinely referred to primary care three months post-AMI. European guidelines recommend a follow-up time in an outpatient setting of at least 8–12 weeks post-AMI while advocating an even more flexible model with a preferred follow-up time of at least one year [11].

EUROASPIRE III and IV which are multinational surveys on CVD prevention, demonstrated that most patients at high CVD risk being treated in the primary care setting do not reach predefined preventive therapeutic and lifestyle goals [4, 24, 29]. Surveys done among primary care physicians in Europe show a lack of use of evidence-based prevention guidelines for reasons like time constraints, lack of perceived usefulness, inadequate knowledge and preference for using own experience [10, 30]. This may partly account for the difference in risk factor control between the two study groups.

The availability and utilization of CR varies. Previous studies, based in the United States, have shown that CR is highly under-utilized, with under half and as low as 13.9% of patients receiving CR [31, 32]. It has also been shown that certain patient groups are significantly less likely to receive CR. These include older individuals and women, with sex-differences increasing with age, as well as, non-whites, and patients with comorbidities (including congestive heart failure, previous stroke, diabetes mellitus, or cancer) [31–33]. However, since Swedish health-care is tax-funded, ensuring equal access to all, irrespective of income, this is less of an issue [34].

### Strengths and limitations

The study was a retrospective observational study where no patient was lost on follow-up. The changes in follow-up structure evaluated in the study were simple and of low cost. They should thus be easy to replicate at other CR centres.

To our advantage, approximately 75% of all Swedish AMI survivors, < 75 years of age, admitted to CCU units around the country, attend CR. However, certain subgroups are overrepresented in the group of non-attenders, especially those with comorbidities, possibly limiting the generalisability of our findings [34].

The study only included AMI survivors under the age of 75 years, thus limiting applicability to other patient and age groups. As there were several differences between tailored and traditional care (i.e. decreased number of cardiologist visits, increased number of telephone contacts, a letter and laboratory measures at 6–8 months and longer time of follow-up) it is impossible to draw any conclusions as to which component of the tailored care resulted in better outcomes.

In our study, patients in the tailored group had a higher participation rate in hospital-based exercise training during follow-up. Exercise training is a core component of a CR programme [11, 17] and it has been shown to lead to improved prognosis and decreased hospital admissions for patients with CVD [35–37]. As such, even though participation in an exercise program was adjusted for in our analysis, a residual confounding effect cannot be ruled out. Also, at the time of the study information on home-based exercise training in the SWEDEHEART registry was not available, causing a possible bias.

Possible external effects such as changes in operations of local primary care centres could have affected the study results, since the groups were divided by a timeline. Possible factors such as behavioural or psychological status, as well as socioeconomic status could have interfered with participation in the CR programme on an individual level. Also, use of ezetimibe increased during the study period, possibly creating a bias even though adjustments were made in the multivariable analyses. In addition, some of the primary end-points were self-reported and were not collected in an otherwise verifiable fashion.

## Conclusion

In conclusion, we showed that providing a tailored, nurse-led CR programme resulted in improvements in lifestyle and risk factor management, supporting the advantages of a tailored, multidisciplinary CR programme.

## Abbreviations

ACEi: Angiotensin converting enzyme inhibitor
AMI: Acute myocardial infarction
ARB: Angiotensin II receptor blocker
BMI: Body mass index
CABG: Coronary artery bypass grafting
CCU: Coronary care unit
CR: Cardiac rehabilitation
CVD: Cardiovascular disease
ESC: European Society of Cardiology
HDL: High density lipoprotein
LDL: Low density lipoprotein
SP: Secondary prevention
SWEDEHEART: The Swedish web-system for Enhancement and Development of Evidence-based care in Heart Disease Evaluated According to Recommended Therapies
